# The impact of FDA and EMA regulatory decision-making process on the access to CFTR modulators for the treatment of cystic fibrosis

**DOI:** 10.1186/s13023-022-02350-5

**Published:** 2022-05-07

**Authors:** Enrico Costa, Silvia Girotti, Francesca Pauro, Hubert G. M. Leufkens, Marco Cipolli

**Affiliations:** 1 WHO Collaborating Centre for Pharmaceutical Policy and Regulations, Utrecht University, Utrecht, The Netherlands; 2 Section of Pharmacology, Department of Diagnostics and Public Health, University of Verona, Verona, Italy; 3 Cystic Fibrosis Center, Azienda Ospedaliera Universitaria Integrata, Verona, Italy; 4 Emeritus Professor Regulatory Science and Pharmaceutical Policy, Utrecht University, Utrecht, The Netherlands

## Abstract

**Background:**

Over the past decade, a new class of drugs called CFTR (cystic fibrosis transmembrane conductance regulator) modulators have shown to be able to improve clinical outcomes in patient with Cystic Fibrosis. In this analysis, we have extensively reviewed the regulatory pathways and decisions adopted by FDA and EMA to speed up the development, the review and the approval of these drugs, with the aim of identifying possible clinical and public health implications associated with differences.

**Results:**

CFTR modulators have been developed towards addressing three main genetic domains: (1) F508del homozygous (F508del/F508del), (2) F508del heterozygous, and (3) genotypes not carrying F508del mutation; and expanded from adult to paediatric population. Programs to expedite the reviewing and licensing of CFTR modulators were extensively adopted by FDA and EMA. All CFTR modulators have been licensed in the US as orphan drugs, but in the EU the orphan status for LUM/IVA was not confirmed at the time of marketing authorization as results from the pivotal trial were not considered clinically significant. While FDA and EMA approved CFTR modulators on the basis of results from phase III double-blind RCTs, main differences were found on the extension of indications: FDA accepted non-clinical evidence considering a recovery of the CFTR function ≥ 10% based on chloride transport, a reliable indicator to correlate with improvement in clinical outcomes. By contrast, EMA did not deem preclinical data sufficient to expand the label of CFTR modulators without confirmatory clinical data.

**Conclusions:**

Regulators played an important role in fostering the development and approval of CFTR modulators. However, differences were found between FDA and EMA in the way of reviewing and licensing CFTR modulators, which extended beyond semantics affecting patients’ eligibility and access: FDA’s approach was more mechanistic/biology-driven while the EMA’s one was more oriented by clinical evidence. This might refer to the connection between the EMA and the Member States, which tends to base decisions on pricing and reimbursement on clinical data rather than pre-clinical ones. Here we have proposed a two-step personalized-based model to merge the ethical commitment of ensuring larger access to all potential eligible patients (including those harboring very rare mutations) with the one of ensuring access to clinically assessed and effective medicines through Real World Data.

**Supplementary Information:**

The online version contains supplementary material available at 10.1186/s13023-022-02350-5.

## Background

Cystic fibrosis (CF) is an autosomal recessive disease caused by mutations in the cystic fibrosis transmembrane conductance regulator (*CFTR*) gene affecting the functional expression of the CFTR protein, an ion channel that regulates the transport of chloride and bicarbonate at the cell surface [1]. Since the discovery of the *CFTR* gene in 1989 [2], more than 2000 mutations have been described, with different prevalence and severity of phenotype. Conventionally, mutations have been grouped into six classes: mutations introducing premature termination codons (i.e. frameshift, splicing, or nonsense mutations) (class I); misfolding mutations (class II); mutations hindering the regulation of the CFTR channel, also known as gating mutations (class III). These classes are usually associated with greater phenotypic severity and worse prognosis. On the other hand, mutations that entail milder clinical symptoms and better prognosis: mutations altering channel conductance (Class IV); mutations reducing the efficiency of CFTR production by affecting splicing (Class V), and mutations reducing the stability of mature CFTR at the cell membrane (Class VI) [3–5].

Early diagnosis and advances in symptomatic therapeutics aimed at dealing with major clinical complications—such as chronic airway infections and pancreatic insufficiency due to an abnormally thick and sticky mucus—have substantially improved the life-expectancy of CF patients [6].

However, better understanding of molecular and cellular pathology of CF paved the way for the development of a new class of drugs—called CFTR modulators—targeting the CFTR function directly [5]. As new agents were developed targeting different genotypes, limitations in the traditional class I–VI CF mutations system became evident and a new more drug-driven approach was developed and adopted by regulators for labelling therapeutic indications [1, 7, 8]. According to the US and the EU orphan legislations, CF is a rare disease and therefore drugs developed for its treatment are potentially eligible for Orphan Drug Designation (ODD), a special status that provides regulatory and financial incentives to sponsors to encourage drug development in unmet medical needs and non-profitable areas. Moreover, the US Food and Drug Administration (FDA) and the European Medicines Agency (EMA) have established similar programmes to expedite the review and approval of medicines to treat serious and unmet medical need conditions such as CF. The aim of this study is to compare FDA and EMA approaches in the evaluation and approval of CFTR modulators and to identify possible clinical and public health implications associated with differences.

## Methods

### Data source and analysis

Qualitative and quantitative data regarding regulatory decisions (i.e. orphan drug designations, expedited programs, and approvals) by FDA and EMA on CFTR modulator medicinal products were retrieved from publicly accessible documents of the Register of FDA Approved Medicines [9] and from the register of medicinal products for human use authorized by the EU under the centralised procedure [10]. Data on evidence supporting regulatory decisions were further searched on the full prescribing information of FDA, the European public assessment report (EPAR) of EMA, clinicaltrials.gov, and published articles. Proofs were categorized into clinical and in vitro studies. Data from clinical studies included: phase and design of the study, eligible population (age and genotype), sample size, primary endpoint(s), outcome(s), and duration of the study/follow-up. Additionally, information on epidemiology and in vitro responsiveness of mutations to CFTR modulators were retrieved from CFTR2 database [11]—a website that provides information for patients, researchers, and the general public about specific variants in what is commonly referred to as the CF gene—and from the FDA and the EMA website, sponsor protocols, and scientific literature as appropriate. All data were updated as of January 30, 2022. Comparative analyses on orphan designations, and expedited programs granted by FDA and EMA were carried out, as well as those on decisions regarding eligible population (age and genotype), timing of decisions, and evidence (clinical studies vs in vitro studies) supporting approvals and variations of indications.

### Classification of the CFTR mutations

Based on the pattern adopted by regulators, mutations have been categorized as follows: F508del, either as homozygous or heterozygous genotype; Gating, as already provided by Class III definition; Conduction, as already provided by Class IV definition; ‘Residual Function (RF)’; Minimal Function (MF); Other, which includes all the mutations not belonging to the above-mentioned categories (see Additional file 1). Acknowledgement of RF and MF mutations hinges upon predicted residual/minimal function of CFTR protein in keeping with population-level phenotypic data and in vitro response to CFTR modulators (IVA and TEZ). MF mutations show a severe phenotype—in fact, they lead to the complete absence of CFTR protein production or function—and do not respond in vitro to either IVA or TEZ, or TEZ/IVA [12, 13]; by contrast, RF mutations have a mild phenotype and respond to the above-mentioned CFTR modulators [14, 15]. Clinical severity has been defined as average sweat chloride (Sweat test—ST) ≥ 86 mmol/L, and prevalence of pancreatic insufficiency (PI) ≥ 50% [13], while in vitro response to CFTR modulators as an increase in percent normal chloride transport of ≥ 10 percentage points to transfected Fischer Rat Thyroid (FRT) cells expressing the CFTR form produced by the mutation [16]. In this study in vitro response data was obtained from assays performed on various cell models, i.e. FRT, CF Bronchial Epithelial (CFBE) and Human Nasal Epithelial (HNE) cell lines.

Published data on the in vitro response to CFTR modulators were searched on *Pubmed* as follows: < name of the mutation > AND < CFTR modulators > or < ivacaftor OR ivacaftor tezacaftor > . When clinical phenotype and in vitro response to CFTR modulators could not be matched to classify the mutation, it was categorized as Other. Two researchers performed the analysis and conflicts were solved through discussion with a third reviewer.

## Results

Four products—developed and marketed by the same company—have been licensed in both the US and the EU: ivacaftor—IVA (Kalydeco®), a potentiator of CFTR function that increases the opening probability of the CFTR channel; lumacaftor/ivacaftor—LUM/IVA (Orkambi®), tezacaftor/ivacaftor—TEZ/IVA (Symdeko® in the US, Symkevi® in the EU), and elexacaftor/tezacaftor/ivacaftor—ELX/TEZ/IVA (Trikafta® in the US, Kaftrio® in the EU), as fixed combinations of potentiators and correctors that address the trafficking through the CFTR protein. All products were first licensed in the US: IVA 5.8 months before the EU approval; LUM/IVA 4.7 months; TEZ/IVA 8.7 months; ELX/TEZ/IVA 10.2 months. In respect of their different pathways, aims and year of launch, expedited programs were extensively adopted by FDA and EMA to foster the reviewing and licensing of CFTR modulators. While all CFTR modulators have been licensed in the US as orphan drugs, in the EU, the EMA’s Committee for Orphan Medicinal Products (COMP) did not recognize the results from the pivotal studies of LUM/IVA (despite statistically significant) as clinically relevant and did not confirm the orphan status at the time of marketing authorization (MA). LUM/IVA therefore missed the 10-year market exclusivity benefit [17]. In the US, expedited programs were implemented long before the development of CFTR modulators, while in the EU IVA and LUM/IVA were developed before PRIority MEdicines (PRIME) scheme came into force.

Anyway, neither in the US nor in the EU, CFTR modulators were considered eligible for earlier approval, as they were not granted accelerated approval by FDA and conditional marketing authorization by EMA respectively (see Additional file 2).

### Expansion of indications

CFTR modulators have been developed towards addressing three main genetic domains: (1) F508del homozygous (F508del/F508del), (2) F508del heterozygous, and (3) genotypes not carrying F508del mutation. In keeping with their own functions and level of responsiveness to CFTR modulators, non-F508del mutations have been clustered in subgroups such as Gating, MF, RF and Other. A few conduction mutations have been described in medical literature, but the only one explicitly reported in approved therapeutic indications was R117H, as it exhibits a gating defect that was partially corrected by IVA.

**Fig. 1:**
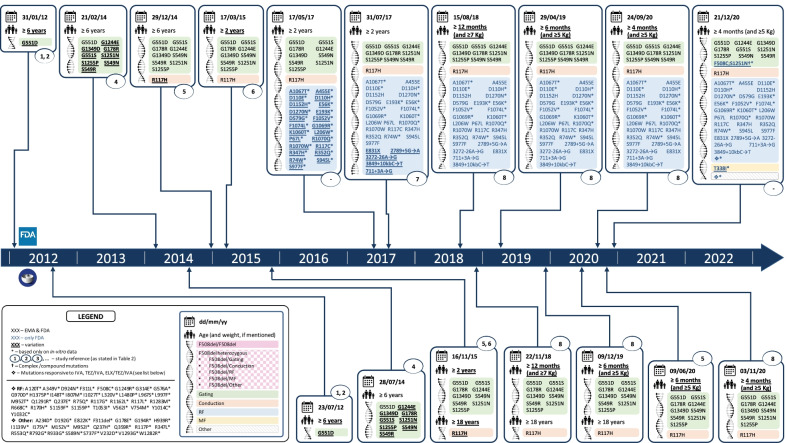
Chronogram of the marketing authorization and the extension of indications of IVA in the US and in the EU. Five extensions of common indications FDA-EMA have been granted by the FDA 5.2 months (1.3–8.1) before the EMA’s approval

**Fig. 2:**
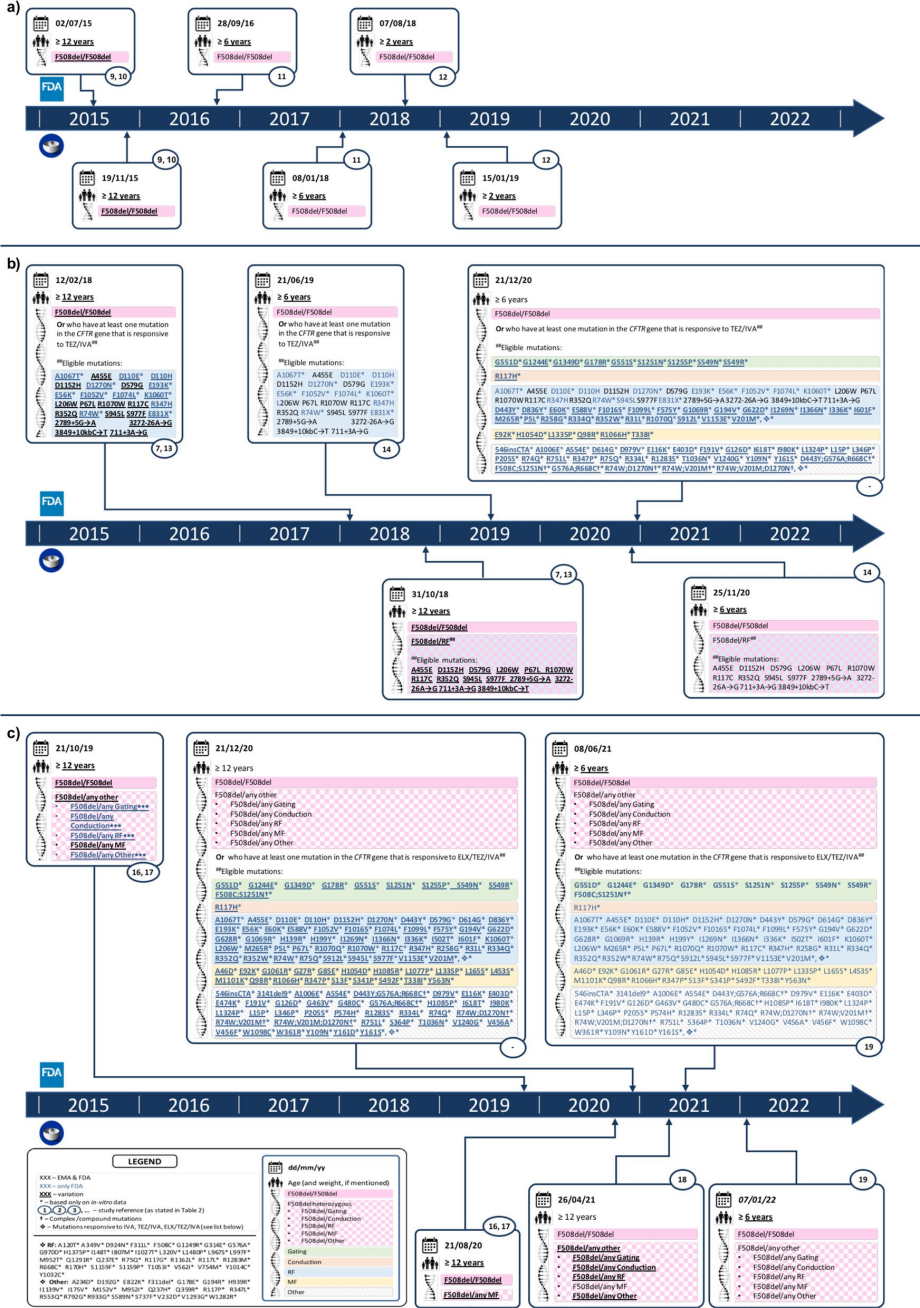
Chronogram of the marketing authorization and the extension of indications of LUM/IVA (**a**), TEZ/IVA (**b**) and ELX/TEZ/IVA (**c**) in the US and in the EU. Extensions of common indications FDA-EMA have been granted first by the FDA: LUM/IVA, 2 extensions in common, median 10.5 months (5.4–15.6); TEZ/IVA, 1 extension in common, 17.4 months before

Over the past 10 years, therapeutic indications have expanded from a limited set to a wider array of mutations, and from individuals aged ≥ 12 years to paediatric population (Figs. 1 and 2). Whenever based on clinical data, FDA and EMA decisions were the same, except for the time delay and the eligible age for IVA of patients carrying the R117H mutation (FDA ≥ 12 years; EMA ≥ 18 years) at the time of licensing [18, 19] and those ≥ 12 years carrying F508del/RF genotype, in which an absolute mean change of + 4.7 (+ 3.7, + 5.8) from baseline in ppFEV_1_ at average of week 4 and 8 led to the approval in the US but not in the EU (VX14-661-108).

Differences, on the other hand, have occurred in those cases where FDA adopted non-clinical evidence for its decision-making process (Table 1). To date, 183 out of 2106 (8.7%) described CF mutations [20] are explicitly eligible for CFTR modulators: 97 for IVA as monotherapy, 154 in combination with TEZ/IVA and 178 with ELX/TEZ/IVA; only 1 for LUM/IVA (F508del, as homozygote).

**Table 1:** Eligible populations and genotypes for the treatments with the currently approved CFTR modulators

		IVA ≥ 4 months (and ≥ 5 kg)		LUM/IVA ≥ 2 years		TEZ/IVA ≥ 6 years		ELX/TEZ/IVA ≥ 6 years	
		FDA	EMA	FDA	EMA	FDA	EMA	FDA	EMA
F508del	F508del	–	–	✓	✓	✓	✓	✓	✓
Gating	F508del	Any Gating mutation responsive to IVA* or **	Any Gating mutation responsive to IVA*	–	–	Any Gating mutation responsive to TEZ/IVA**	–	✓	✓
	Non-F508del							Any Gating mutation responsive to ELX/TEZ/IVA**	–
Conduction	F508del	R117H or any Conduction mutation responsive to IVA**	R117H	–	–	Any Conduction mutation responsive to TEZ/IVA**	–	✓	✓
	Non-F508del							Any Conduction mutation responsive to ELX/TEZ/IVA**	–
RF	F508del	Any RF mutation responsive to IVA* or **	–	–	–	Any RF mutation responsive to TEZ/IVA* or **	Any RF mutation responsive to TEZ/IVA*	✓	✓
	Non-F508del						–	Any RF mutation responsive to ELX/TEZ/IVA**	–
MF	F508del	Any MF mutation responsive to IVA**	–	–	–	Any MF mutation responsive to TEZ/IVA**	–	✓	✓
	Non-F508del							Any MF mutation responsive to ELX/TEZ/IVA**	–
Other	F508del	Any Other mutation responsive to IVA**	–	–	–	Any Other mutation responsive to TEZ/IVA**	–	✓	✓
	Non-F508del							Any Other mutation responsive to ELX/TEZ/IVA**	–

(The complete list of each single eligible mutation is reported in Figs. 1 and 2) Definitions: (✓) = eligible; (–) = not eligible; *based on clinical evidence; **based only on in vitro data

*IVA* ivacaftor (VX-770), *LUM* lumacaftor (VX-809), *TEZ* tezacaftor (VX-661), *ELX* elexacaftor (VX-445), *RF* residual function CFTR mutation, *MF* minimal function CFTR mutation

### Clinical evidence

CFTR modulators have been licensed on the basis of results from phase III double-blind RCTs, whereas extensions of indications relied either on RCTs or on open-label single-group studies. Two studies regarding ELX/TEZ/IVA in patients ≥ 12 years F508del/F508del (VX17-445-03) and ≥ 12 years F508del/RF or F508del/Gating (VX18-445-04), provided an active comparator. Three studies—all regarding extensions of indication for IVA in patients 2–5 years and < 24 months patients harbouring Gating mutations, and LUM/IVA in patients 2–5 years F508del/F508del—were open-label single group CTs. The main primary endpoint adopted has been the absolute mean change from baseline in ppFEV_1_; however, in populations aged 6–11 years the lung clearance index (LCI_2.5_) was acknowledged as a more a sensitive measure of ventilation inhomogeneity, since it is able to detect early peripheral airway damage in CF patients with a greater sensitivity than spirometry. The two Agencies made consistent decisions on refusals, which regarded a phase II RCT (VX08-770-104) aimed at extending the indications of IVA to patients ≥ 12 years F508del/F508del and a phase III RCT (VX14-661-107) for the extension of TEZ/IVA to patients ≥ 12 years old F508del/MF, with an absolute mean change respectively of + 1.7 from baseline in ppFEV_1_ through week 16, and + 1.2 through week 12 (Table 2).

**Table 2:** Clinical trials supporting regulatory decisions for the Marketing Authorization and the extensions of indication of CFTR modulators

	FDA	EMA	Phase	Study	Control	Study Population	Treatment Duration	Primary Endpoint	Primary Outcome	Ref
*IVA—Kalydeco*										
1	31/01/12	23/07/12	III	VX08-770-102 (NCT00909532) STRIVE	Placebo (parallel)	≥ 12 years—G551D Sample size: 161	48 weeks	Absolute mean change from baseline in ppFEV_1_ through wk 24	LS mean absolute change IVA vs placebo (95% CI): + **10.6 (+ 8.6, + 12.6)**	[36]
2			III	VX08-770-103 (NCT00909727) ENVISION	Placebo (parallel)	6 to 11 years—G551D Sample size: 52	48 weeks	Absolute mean change from baseline in ppFEV_1_ through wk 24	LS mean absolute change IVA vs placebo (95% CI): + **12.5 (+ 6.6, + 18.3)**	[57]
3	Not approved	Not approved	II	VX08-770-104 (NCT00953706) DISCOVER	Placebo (parallel)	≥ 12 years—F508del/F508del Sample size: 140	16 weeks	Aabsolute mean change from baseline in ppFEV_1_ through wk 16	LS mean absolute change IVA vs placebo (95% CI): + **1.7 (-0.6, + 4.1)**	[58]
4	21/02/14	28/07/14	III	VX12-770-111 (NCT01614470) KONNECTION	Part A: Placebo (crossover) Part B: open-label	≥ 6 years—non-G551D gating mutation (*) Sample size: 39 (Part A: 39 Part B: 36)	24 weeks Part A: 8 wk Part B: 16 wk extension	(A) absolute change from baseline in ppFEV_1_ through wk 8	(A) LS mean difference IVA vs placebo (95% CI): + **10.7 (+ 7.3, + 14.1)**	[32]
								(B) Absolute change from baseline in ppFEV_1_ through wk 24	(B) LS mean absolute change IVA vs placebo (95% CI): + **13.5 (-6.9, + 36.5)**	
5	29/12/14	16/11/15 09/06/20	III	VX11-770-110 (NCT01614457) KONDUCT	Placebo (parallel)	≥ 6 years—R117H, non-gating mutation Sample size: 69	24 weeks	Absolute change from baseline in ppFEV_1_ through wk 24	LS mean difference IVA vs placebo (95% CI): + **2.11 (-1.13, + 5.35)**	[33]
6	17/03/15	16/11/15	III	VX11-770-108 (NCT01705145) KIWI	Open-label	2 to 5 years—gating mutation (G551D, *) Sample size: 34 (Part A: 9 Part B: 33)	Part A: 4 days Part B: 24 weeks	(A) pharmacokinetic (A, B) safety: number of participants with AEs, SAEs and related AEs	(A) safety: 8 subj had AEs (88.9%), no SAEs, 4 subj (44.4%) had related AEs (B) safety: 33 subj had AEs (97.1%), 6 subj (17.6%) had SAEs, 11 subj had related AEs (32.4%)	[59]
7	31/07/17	Not approved	III	VX14-661108 (NCT02392234) EXPAND	Placebo (crossover)	≥ 12 years—F508del/RF Sample size: 244	8 weeks crossover	Absolute change from baseline in ppFEV_1_ at average of wk 4 and 8	LS mean difference IVA vs placebo (95% CI): + **4.7 (+ 3.7, + 5.8)**	[14]
8	15/08/18	22/11/18	III	VX15-770-124 (NCT02725567) ARRIVAL	Open-label	< 24 months—gating mutation (G551D, *) or R117H Sample size: Part A (Cohort 1): 7 Part B (Cohort 5): 19	Part A: 4 days Part B: 24 weeks	(A) pharmacokinetic (A, B) safety: number of participants with AEs, SAEs and related AEs	(A) safety: 3 subj had AEs (42.9%), no SAEs, no related AEs (B) safety: 18 subj had AEs (94.7%), 2 subj (10.5%) had SAEs, 7 subj (36.8%) had related AEs	[60]
	29/04/19	09/12/19			Open-label	< 24 months—gating mutation (G551D, *) or R117H Sample size: Part A (Cohort 2): 6 Part B (Cohort 6): 11			(A) safety: 4 subj had AEs (66.7%), no SAEs (B) safety: 10 subj had AEs (90.9%), 3 subj (27.3%) had SAEs, 2 subj (18.2%) had related AEs	[61]
	24/09/20	03/11/20			Open-label	< 24 months—gating mutation (G551D, *) or R117H Sample size: Part A (Cohort 3): 6 Part B (Cohort 7): 6			(A) safety: 3 subj had AEs (50.0%), 1 subj (16.7%) had SAEs, no related AEs (B) safety: 6 subj had AEs (100%), 1 subj (16.7%) had SAEs, no related AEs	[61]
*LUM/IVA—Orkambi*										
9	02/07/15	19/11/15	III	VX12-809-103 (NCT01807923) TRAFFIC	Placebo (parallel)	≥ 12 years—F508del/F508del Sample size: 549	24 weeks	Absolute change from baseline in ppFEV_1_ through wk 24	LS mean difference LUM/IVA vs placebo (pooled analysis) (95% CI): + **3.3 (+ 2.3, + 4.3)** for LUM 600 mg + **2.8 (+ 1.8, + 3.8)** for LUM 400 mg	[62]
10				VX12-809-104 (NCT01807949) TRANSPORT	Placebo (parallel)	≥ 12 years—F508del/F508del Sample size: 559	24 weeks			
11	28/09/16	08/01/18	III	VX14-809-109 (NCT02514473)	Placebo (parallel)	6 to 11 years—F508del/F508del Sample size: 204	24 weeks	Absolute change in LCI_2.5_ through wk 24	LS mean LUM/IVA vs placebo (95% CI): − **1.1 (− 1.4, − 0.8)**	[39]
12	07/08/18	15/01/19	III	VX15-809-115 (NCT02797132)	Open-label	2 to 5 years—F508del/F508del Sample size: 62 (Part A: 12 Part B: 60)	Part A: 15 days Part B: 24 weeks	(A) pharmacokinetic (A, B) safety: number of participants with AEs and/SAEs	(B) safety: 59 subj had AEs (98%), 4 subj (7%) had SAEs	[63]
*TEZ/IVA—Symdeko (Symkevi)*										
13	12/02/18	31/10/18	III	VX14-661-106 (NCT02347657) EVOLVE	Placebo (parallel)	≥ 12 years—F508del/F508del Sample size: 504	24 weeks	Absolute change from baseline in ppFEV_1_ through wk 24	LS mean difference TEZ/IVA vs placebo (95% CI): + **4.0 (+ 3.1, + 4.8)**	[64]
7			III	VX14-661-108 (NCT02392234) EXPAND	Placebo (crossover)	≥ 12 years—F508del/RF Sample size: 244	8 weeks	Absolute change from baseline in ppFEV_1_ at average of wk 4 and 8	LS mean difference TEZ/IVA vs placebo (95% CI): + **6.8 (+ 5.7, + 7.8)**	[14]
14	21/06/19	25/11/20	III	VX16-661-115 (NCT03559062)	Placebo (parallel)	6 to 11 years—F508del/RF + F508del/F508del Sample size: 67	8 weeks	Absolute change in LCI_2.5_ through wk 8	LS mean TEZ/IVA vs placebo (95% CI): − **0.51 (-0.74, − 0.29)**	[65]
15	Not approved	Not approved	III	VX14-661-107 (NCT02516410)	Placebo (parallel)	≥ 12 years—F508del/MF Sample size: 168	12 weeks	Absolute change from baseline in ppFEV_1_ through wk 12	LS mean difference TEZ/IVA vs placebo (95% CI): + **1.2 (-0.3, + 2.6)**	[12]
*ELX/TEZ/IVA—Trikafta (Kaftrio)*										
16	21/10/19	21/08/20	III	VX17-445-102 (NCT03525444)	Placebo (parallel)	≥ 12 years—F508del/MF Sample size: 403	24 weeks	Absolute change in ppFEV_1_ from baseline at wk 4	LS Mean difference ELX/TEZ/IVA vs control (95% CI): + **13.8 (+ 12.1, + 15.4)**	[34]
17			III	VX17-445-103 (NCT03525548)	Active (parallel)	≥ 12 years—F508del/F508del Sample size: 107	4 weeks	Absolute change in ppFEV_1_ from baseline at wk 4	LS Mean difference ELX/TEZ/IVA vs control (95% CI): + **10 (+ 7.4, + 12.6)**	[66]
18	N/A	26/04/21	III	VX18-445-104 (NCT04058353)	Active (parallel)	≥ 12 years—F508del/RF + F508del/Gating Sample size: 258	8 weeks	Absolute change in ppFEV_1_ from baseline at wk 8	LS Mean difference ELX/TEZ/IVA vs control (95% CI): + **3.7 (+ 2.8, + 4.6)**	[35]
19	08/06/21	07/01/22	III	VX18-445-106 (NCT03691779)	Open-label	6 to 11 years—F508del/MF + F508del/F508del Sample size: 66 (Part A: 16 Part B: 66)	Part A: 15 days Part B: 24 weeks	(A) pharmacokynetic (A, B) safety: number of participants with TEAEs and SAEs	(A) safety: 12 subj had AEs (75%), no SAEs (B) safety: 65 subj had AEs (98.5%), 1 subj (1.5%) had SAEs	[67]

Bold style has been used only to highlight the results of the studies

Definitions: * = G178R, S549N, S549R, G551S, G970R, G1244E, S1251N, S1255P or G1349D; RF = residual function *CFTR* mutation according to the clinical trial list [14]; *MF* minimal function *CFTR* mutation according to the clinical trial list [68], *IVA* ivacaftor (VX-770), *LUM* lumacaftor (VX-809), *TEZ* tezacaftor (VX-661), *ELX* elexacaftor (VX-445), *RCT* Randomized Controlled Trial; SID = once daily, *BID* twice daily, *ppFEV*_*1*_ percentage of predicted forced expiratory volume in 1 s, *LCI*_*2.5*_ Lung Clearance Index 2.5: its decrease indicates improvement in lung function; *AEs* Adverse Events, *SAEs* Serious Adverse Events, *LS Means* Least Squares Means, *CI* confidence interval

### Non-clinical evidence

FDA has accepted in vitro studies for extending indications. Non-clinical endpoints taken into account include: a) the total ionic current (IT) due to the cell surface channel density; b) gating activity and conductance applied to FRT cells harbouring the mutation under study; c) the open channel probability (PO), representing the time interval when a single CFTR protein channel is open and transports ions; d) CFTR maturation, which relies on Western blotting techniques and monitors the cellular trafficking of CFTR to the apical surface. A recovery of the CFTR function ≥ 10% based on chloride transport has been considered reliable to lead to milder clinical manifestations of CF, i.e. a lower incidence of pancreatic insufficiency, and a more moderate lung function decline and lower sweat chloride levels, compared to patients with minimal CFTR chloride transport [21]. In 2017, after a previous rejection in 2016 [22], based on data from in vitro cell-based assays and on results from a previously exploratory phase IIa study, FDA granted 23 RF mutations as eligible for IVA [23, 24]. On the contrary, 26 MF mutations—not meeting the chloride transport threshold—were not approved [25]. As a post-marketing commitment, the Sponsor was required to conduct a 3-year single arm, observational study to further understand the clinical response to IVA in different subgroups of CF patients with CFTR mutations deemed responsive to IVA based on in vitro evidence [26]. Moreover, upon the same type of assays, mutation T338I—which had previously been refused—was approved for IVA (Fig. 1), TEZ/IVA and ELX/TEZ/IVA (Fig. 2) [27]. Overall, 82 mutations have been approved for IVA on the basis of in vitro assays. Following on the granting of RF mutations for IVA, in vitro assays were applied to other CFTR modulators.

TEZ/IVA was first licensed in the US on the basis of clinical evidence from in vitro studies. FRT assays were conducted on genotypes carrying IVA-responsive mutations (already approved in 2017) and F508del. TEZ/IVA showed a similar—rather than an increased—chloride transport level in comparison with IVA. However, any correlations of clinical benefit over IVA remained unclear. Subsequently, one of the two pivotal CTs—the crossover 3-treatment EXPAND study—confirmed the correlation of in vitro response to clinical efficacy of TEZ/IVA for 16 RF mutations. After the extension to patients ≥ 6 years, FDA approved an additional set of mutations through this pathway: in 2020, 127 additional mutations were granted eligibility for TEZ/IVA—quite surprisingly, 6 of these mutations (MF) were approved despite not being responsive in vitro—and 177 for ELX/TEZ/IVA (Fig. 2).

## Discussion

CFTR modulators have been a groundbreaking and unprecedent achievement for CF. These small-molecules were first discovered through high-throughput screening (HTS) with medicinal chemistry interventions driven by predictive in vitro assays, and then brought into CTs. All CFTR modulators were first launched on the US market, which is the most remunerative pharmaceutical market in the world [28]. However, this might also refer to the fact that the sponsor is a US-based company and the initial development of CFTR modulators was supported by the US CF Foundation (CFF), thus, exerting some sort of pressure on the company's marketing strategy and policy [29]. Differences were also found in regulatory approaches, partly due to the legal background of the two systems, partly related to scientific principles, culture of weighing benefits and risks, and dealing with the subsequent Health Technology Assessment (HTA) evaluations [30]. For example, COMP’s decision on confirmation of the ODD for LUM/IVA at the time of MA steered Member States’ assessment on value-based pricing and reimbursement: HTA organizations emphasized that a < 4% change in FEV_1_ was not a relevant clinical outcome, since its correlation to pulmonary exacerbations (PEX) or to other clinically relevant outcomes remains unclear [31].

### Development strategy for CFTR modulators

The clinical development of CFTR modulators expanded from the monotherapy of the potentiator IVA in a selected group of Gating mutations[32, 33], to the last combination ELX/TEZ/IVA [21] targeting the CFTR function in patients carrying at least one F508del mutation [34, 35]. The overarching approach adopted for studying CFTR modulators in clinical stage was short-term double-blind RCTs followed by open label long-term safety studies [36, 37]. Meanwhile, investigations moved towards the paediatric population, providing extrapolated data from older population as well as assessing pharmacokinetics, safety and tolerability [38, 39]. Efficacy has been found heterogeneous across different CFTR modulators and targeted population, but—remarkably—FDA and EMA adopted the same decisions for both approvals or refusals.

Results from IVA targeting G551D mutation [36] were considered the benchmark for the development of following CFTR modulators: accordingly, LUM/IVA and TEZ/IVA showed a modest clinical benefit, while ELX/TEZ/IVA was recognized as the standard of care for genotypes carrying at least one F508del mutation, namely the most frequent allelic variant in CF: with slight geographical differences, current CFTR modulators could target on average 70–80% of CF patients [40, 41]. By way of contrast, 20–30% of patients are not yet eligible for treatments, and this percentage includes: a) rare mutations neither enrolled in CTs nor studied, and therefore not licensed by regulators; b) mutations whose biological features prevent the use of CFTR modulators, i.e. premature stop mutations producing truncated unstable mRNA and a lack of full-length CFTR proteins, so that the use of CFTR modulators would be not plausible.

### Predictable models

The extrapolation of data from preclinical models to expand the clinical use of CFTR modulators has been the major difference found between FDA and EMA [42]. As ion transport properties of primary human CF respiratory epithelial cells can be preserved in cell cultures, non-clinical studies have been used as proof-of-concept to demonstrate the preliminary efficacy of CFTR modulators [43].

Testing modulators on a variety of laboratory or patient-derived cells (Theratyping) has the potential of characterizing complex CFTR variants, of assessing modulator responsiveness of rare/unique CFTR mutations, and even of providing an optimization in the modulator therapy regimen through modulator responsiveness comparison. Patient-derived model systems may avoid the challenges of varying responses to CFTR modulators within the same genotype among different patients (for the purpose of a personalized therapy) and can support the selection of suitable “likely responders to drug” subgroups to be enrolled in CTs through the characterization of unclassified CFTR variants by the response to modulators [44]. For example, a strong correlation between in vitro data and clinical outcomes has been observed with IVA in patients carrying F508del mutation: < 10% recovery of CFTR was subsequently confirmed as a non-statistically significant increase for FEV_1_. Although stringent criteria must be met before considering the use of in vitro data alone to expand a drug indication—such as a good understanding of the disease and a solid comprehension of the drug’s mechanism of action [45]—not all situations have confirmed such correlation: LUM/IVA showed a very promising 25.1% recovery of CFTR functionality in F508del genotypes but a modest increment in FEV_1_ [46]. These discrepancies have raised doubts on the validity of preclinical models and their use for regulatory approvals. In the EU, preclinical data were accepted for granting initial ODD, a stage where non-clinical studies were considered reliable for anticipating clinical effects of new products. By contrast, at the time of MA only confirmed clinical data were acknowledged.

### Implications for patients and healthcare systems

Differences between FDA and EMA in the way of reviewing and licensing medicines lay on procedures and relevant clinical decisions. In oncology, for example, the Agency that provided a positive opinion was found to be more restrictive in terms of wording indications compared with the Agency that first granted approval [47]. In the case of CF, differences extend beyond semantics, procedures and timing of approval, but affect patients’ eligibility. In the US, extensions of indication based on in vitro data have addressed patients carrying mutations not included in CTs because of their rarity [48]. Since then, new and alternative predictable models have been implemented. Recent advances in adult stem cell biology have produced the development of organoids using a variety of tissue sources such as intestine, respiratory epithelium and kidney [48]. When no approved treatments are available for rare mutations, and large scale CTs are therefore not feasible, n-of-1 trials have been proposed to contribute to the totality of evidence for expanding drug indications. However, this approach has some methodological strengths and weaknesses to be carefully considered before supporting expansion of access to expensive medicines [49].

### A stepwise model to merge drug-regulation and HTA

As regulators have to manage the challenge of uncertainty in the benefit/harm assessment, systems of personalized therapy might progressively support regulatory decisions and subsequent HTA evaluations. An increased coordination between these two levels may promote a new and more flexible model that could fall under the tag of ‘payment at results’ agreements. The introduction of Next-Generation Sequencing in clinical practice has opened new perspective for precision and personalized medicines. In oncology, the need for systematic interpretation of molecular alterations and their translation into clinical practice has been addressed thorough the implementation of the so-called ‘Molecular Tumour Boards’: a panel of experts who analyse tumour genotypes in order to recommend the most suitable targeted therapy [50, 51]. A ‘CF Molecular Board’ could be implemented, with a view to promoting an efficient and timely manner access to CFTR modulators to patient carrying mutations not included in CTs. Genotypes might be screened and when considered eligible for CFTR modulators, they should be candidate to theratyping tests, the results of which should be confirmed in clinical setting. Efficient use is essential for public health funded systems, in particular in the case of orphan drugs which are tagged at high nominal price [52]. This approach can meet both the ethical imperative of taking care of individual patients and the recognition of a value-based price.

### Reviewing pharmaceutical R&D funding for rare diseases

Despite the unquestionable advances in the treatment of CF and the potential impact of personalized approaches to target further rare mutations, a significant number of individuals in the world do not benefit from CFTR modulators. And there is more to come. New CFTR modulators and new innovative approaches are currently in development to target patients who have experienced limited benefits from already approved CFTR modulators and also for targeting non-sense mutations [53].

Orphan legislations and incentive systems have brought a huge contribution to target unmet medical needs, also in CF. But it is time now to rethink and set sustainable policies for the future, by ensuring R&D programs to meet patients’ needs, as well as equitable access to the innovations. In the US, new approaches have been recently implemented on public–private collaboration to foster the delivery of new gene therapies to patients affected by ultra-rare diseases [54].

However, also in the EU, one of the four pillars of the ‘Pharmaceutical strategy for Europe’ aims at ensuring access to affordable medicines for patients, and at addressing unmet medical needs, such as CF [55].

In the area of (ultra)rare diseases, experimenting with public–private partnerships throughout the life-cycle of a drug could better address its development towards medical needs, so mitigating and sharing business risks and dealing with failures, and most importantly steering the pricing of drugs once they are placed on the market, in order to improve their affordability and subsequent access for patients.

### Public and global health outlook

The epidemiological profile of CF has been changing. Advances in the management of the disease have increasingly transformed what was considered an exclusive pediatric disease into an adult disorder. On the other hand, epidemiological studies have shown that CF extend beyond the US and the EU boundaries. Given its higher prevalence among Caucasians, CF has long been considered an exclusive disease of western countries. However, data on CFTR mutations have been progressively reported from Asia, the Middle East, Latin America and Africa [56]. The incidence of CF in Low-Income Countries (LICs) is variable and depends on the composition and origin of the population, and the awareness of the condition which inevitably leads to its underdiagnosis, misdiagnosis, and underreporting. However, nowadays effective medicines are available and their patents are expiring, which can globally lead to an improvement of their affordability. Meanwhile, confirmatory results from clinical use of CFTR modulators on rare mutations might also contribute to maximizing the cost–benefit profile of these medicines in LICs. But in Western Countries several challenges remain, especially for HTA where comparative CTs and the contribution of Real-World Evidence (RWE) are expected to increasingly contribute to better define the place in therapy of different treatments and their value (and hence their accessibility).

## Conclusions

Our analysis has brought valuable insights on the regulatory decision-making process of FDA and EMA on CFTR modulators for the treatment of CF, emphasizing the role of regulators in fostering the development and approval of these medicines and the streamlined access to a growing number of patients. Remarkably, FDA took the unusual decision of expanding the use of CFTR modulators on the basis of data from in vitro. By contrast, EMA did not deem preclinical data sufficient to expand the label of CFTR modulators without clinical data.

Such differences raise an important question: what should drive the approval of new drugs or a new indication? Clinical evidence or biological markers? We proposed a two-step personalized-based model to merge the ethical commitment of ensuring larger access to all potential eligible patients (as provided by FDA) with the one of ensuring access to clinically assessed and effective medicines (as provided by EMA).

As most of the novel medicines that have been introduced in clinical practice globally are first approved by FDA and EMA, the two-step approach we have proposed here—to confirm biological plausibility in clinical practice within a reimbursement agreement—can provide a more comprehensive amount of knowledge for an incremental cost-effective use of CFTR modulators worldwide.

## Supplementary Information

**Additional file 1.** Algorithm adopted to classify the eligible mutations to CFTR modulators as approved by FDA and EMA. Complete phenotypic data comprehends: average sweat chloride (sweat test, ST) (mmol/L) and pancreatic insufficiency in percentage (PI%). Criteria for severe phenotype are ST ≥ 86 mmol/L, and PI% ≥ 50%. Definitions: * = if mentioned; WT-CFTR = wild-type CFTR protein; RF = residual function CFTR mutation; MF = minimal function CFTR mutation. Other: a = Uncomplete/missing phenotypic data; b = conflicting phenotypic; c = conflicting phenotypic/responsiveness data.

**Additional file 2.** Framework for fostering the development, review and approval of medicines for rare and serious life-threatening conditions in the US and in the EU. Definitions: MA = Marketing Authorization, SMEs = small & medium-sized enterprises. At the time of marketing authorization LUM/IVA was withdrawn from the Community Register of designated Orphan Medicinal Products of the EU upon request of the sponsor [25]. In the EU, the designation to accelerated assessment - which shortens the review time from 210 to 150 days - was granted to IVA and LUM/IVA, while the EMA did not agree to the applicant’s request for TEZ/IVA being considered not of major public health interest [69]. The triple combination ELX/TEZ/IVA was initially reviewed under EMA’s accelerated assessment program, but since the applicant requested a 3-month clock stop during assessment - ultimately reduced to 2 months - the conditions for accelerated assessment could no longer be met [35].

## Data Availability

Please contact author for data requests.
