# Supplementary figures and images for: The impact of FDA and EMA regulatory decision-making process on the access to CFTR modulators for the treatment of cystic fibrosis
Source: Orphanet J Rare Dis. 2022 May 7;17:188. doi: 10.1186/s13023-022-02350-5 (PMC9078013; doi:10.1186/s13023-022-02350-5)

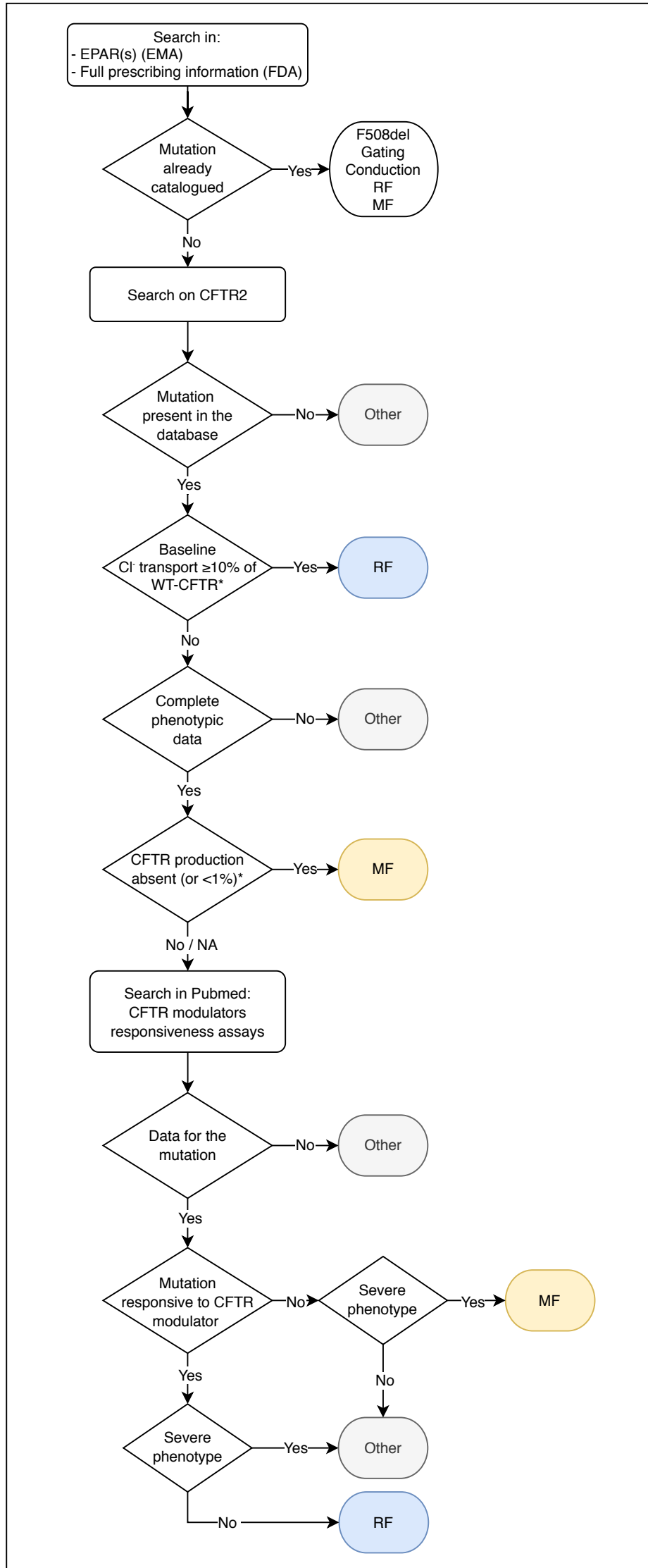

Supplement: Supplementary file 1 — Additional file 1. Algorithm adopted to classify the eligible mutations to CFTR modulators as approved by FDA and EMA. Complete phenotypic data comprehends: average sweat chloride (sweat test, ST) (mmol/L) and pancreatic insufficiency in percentage (PI%). Criteria for severe phenotype are ST ≥ 86 mmol/L, and PI% ≥ 50%. Definitions: * = if mentioned; WT-CFTR = wild-type CFTR protein; RF = residual function CFTR mutation; MF = minimal function CFTR mutation. Other: a = Uncomplete/missing phenotypic data; b = conflicting phenotypic; c = conflicting phenotypic/responsiveness data. [file 13023_2022_2350_MOESM1_ESM.pdf]
