# Supplementary material for: The impact of FDA and EMA regulatory decision-making process on the access to CFTR modulators for the treatment of cystic fibrosis
Source: Orphanet J Rare Dis. 2022 May 7;17:188. doi: 10.1186/s13023-022-02350-5 (PMC9078013; doi:10.1186/s13023-022-02350-5)
Supplement: Supplementary file 2 — Additional file 2. Framework for fostering the development, review and approval of medicines for rare and serious life-threatening conditions in the US and in the EU. Definitions: MA = Marketing Authorization, SMEs = small & medium-sized enterprises. At the time of marketing authorization LUM/IVA was withdrawn from the Community Register of designated Orphan Medicinal Products of the EU upon request of the sponsor [25]. In the EU, the designation to accelerated assessment - which shortens the review time from 210 to 150 days - was granted to IVA and LUM/IVA, while the EMA did not agree to the applicant’s request for TEZ/IVA being considered not of major public health interest [69]. The triple combination ELX/TEZ/IVA was initially reviewed under EMA’s accelerated assessment program, but since the applicant requested a 3-month clock stop during assessment - ultimately reduced to 2 months - the conditions for accelerated assessment could no longer be met [35]. [file 13023_2022_2350_MOESM2_ESM.pdf]

Agency	Year	Category	Program	Qualifying criteria	Program benefits	IVA	LUM/IVA	TEZ/IVA	ELX/TEZ/IVA
FDA	1983	designation	Orphan Drug Designation	Treatments for diseases or conditions affecting <200,000 persons in the US OR drugs that will not be profitable within 7 years following approval by the FDA	Tax credits for qualified clinical testing, waiver of NDA/BLA user fees, eligibility for 7-year marketing exclusivity upon MA	✅	✅	✅	✅
	1992	designation	Priority Review	Significant improvement in safety or efficacy for the treatment, diagnosis, or prevention of seriuos conditions compared to standard applications	Shorter FDA review timeframe: 6 months vs standard 10 months	✅	✅	✅	✅
	1992	approval pathway	Accelerated Approval	Meaningful advantage over available therapies and demonstrated effect on surrogate endpoint reasonably likely to predict clinical benefit	Approval based on surrogate endpoint or intermediate clinical endpoint (not irreversible morbidity or mortality)	❌	❌	❌	❌
	1997	designation	Fast-Track	Potential to address serious conditions and fill an unmet medical need, on the basis of preclinical data	Actions to expedite development process and review: frequent interactions with FDA during drug development, rolling review	✅	✅	✅	✅
	2012	designation	Breakthrough Therapy	Treatment of serious or life-threatening diseases AND substantial improvement on a clinically significant endpoint over available therapies	Intensive actions to expedite the drug development process: all Fast-Track program features, intensive FDA guidance, organizational commitment involving senior managers	✅	✅	✅	✅
EMA	2000	designation	Orphan Drug Designation	Significant benefit for life-threatening or chronically debilitating diseases affecting <5 in 10,000 in the EU OR medicines that will not provide sufficient returns from marketing to justify its development investment	Protocol assistance, fee reduction, centralized MA, incentives for SMEs, eligibility for 10-year marketing exclusivity upon MA	✅	⚠️	✅	✅
	2004	designation	Accelerated Assessment	Major interest for public health, particularly in the therapeutic innovation field	Shorter EMA review timeframe: 150 days vs standard 210 days	✅	✅	❌	⚠️
	2004	approval pathway	Authorisation under Exceptional Circumstances	Unability to provide comprehensive efficacy and safety data under normal conditions of use; the condition to be treated is rare* or the collection of full information is not possible or unethical	Less comprehensive evidence at time of MA compared with normal requirement	❌	❌	❌	❌
	2006	approval pathway	Conditional Marketing Authorisation	Benefit to public health of immediate availability outweighs risk of less comprehensive data than usual	Less comprehensive evidence at the time of MA compared with normal requirement**	❌	❌	❌	❌
	2016	designation	PRIME – PRIority MEdicines	Major therapeutic advantage over existing treatments, or fulfillment of an unmet medical need; potential to benefit patients with unmet medical needs assessed on the basis of early clinical data.	Early and proactive support of the EMA for the development, possible eligibility to accelerated assessment applications	NA	NA	❌	❌

NA = program not available at the time of drug evalutation ✅ = included in the program ❌ = not included in the program ⚠️ = first included, subsequently withdrawn

* = Orphan designation criteria are independent from the criteria to be considered for approval under exceptional circumstances ** = the applicant should be in a position to provide the comprehensive clinical data in a short timeframe
